# Birth of Thirty-Two Healthy Babies Following Transfer of Fresh and Frozen–Thawed Embryos Derived from Monopronuclear Zygotes: A Retrospective Study

**DOI:** 10.3390/medicina60081361

**Published:** 2024-08-21

**Authors:** Soraya Labied, Frédéric Wenders, Olivier Gaspard, Stéphanie Ravet, Alice Desmecht, Michelle Nisolle, Laurie Henry

**Affiliations:** 1 Center for Reproductive Medicine, University of Liège, Boulevard du 12ème de Ligne 1, 4000 Liege, Belgium; frederic.wenders@citadelle.be (F.W.); olivier.gaspard@citadelle.be (O.G.); stephanie.ravet@citadelle.be (S.R.); 2 Faculty of Medicine, University of Liège, Place du Vingt Août 7, 4000 Liege, Belgium; alice.desmecht@student.uliege.be; 3 Obstetrics and Gynecology Department, University of Liège, Boulevard du 12ème de Ligne 1, 4000 Liege, Belgium; michelle.nisolle@citadelle.be

**Keywords:** monopronuclear, embryo transfer, live birth, in vitro fertilization

## Abstract

*Background and Objectives:* Fertilized zygotes normally display two pronuclei (PN), but abnormal fertilization patterns (0, 1 or >2PN) are observed daily in IVF labs. Multiple PN zygotes (>2) are generally discarded due to an increased risk of aneuploidy. However, the decision to transfer or not transfer 1PN-derived embryos remains controversial. The aims of our study were to analyze the neonatal outcomes of fresh or frozen–thawed embryos derived from 1PN zygotes, and to evaluate the influence of the fertilization method. *Materials and Methods*: Data were retrospectively collected from cycles performed between January 2018 and December 2022. Fresh cycles were analyzed for the comparative fate of 1PN zygotes (n = 1234) following conventional in vitro fertilization (cIVF; n = 648) or intracytoplasmic sperm injection (ICSI; n = 586), as well as the results of the 64 transfers of 1PN-derived embryos (pregnancy rate (PR) and neonatal outcomes). This pregnancy follow-up was also applied to 167 transfers of frozen–thawed 1PN-derived embryos. *Results:* In fresh cycles, 46% of the 1PN zygotes in the cIVF group developed into embryos of sufficient quality to be transferred or frozen (day 3 or 5/6). This rate was lower in the fresh ICSI cycles (33%). Blastulation rate was also significantly higher in the cIVF group (44%) in comparison to the ICSI group (20%). The fresh single embryo transfers (32 per group) allowed seven pregnancies in the cIVF group (PR = 21.9%) as compared to four pregnancies in the ICSI group (PR = 12.5%). In the cIVF group, five deliveries of healthy newborns were achieved, but only one in the ICSI group. In frozen/thawed cycles, 36 pregnancies were obtained out of the 167 transfers. A non-significant difference was observed between embryos derived from cIVF cycles (PR = 26%) and ICSI cycles (PR = 16%) with 18 and 8 healthy babies born, respectively. *Conclusions:* We observed better outcomes for 1PN zygotes in cIVF cycles in comparison to ICSI cycles. Our center policy to transfer good-quality 1PN-derived embryos allowed the birth of 32 healthy babies.

## 1. Introduction

In assisted reproduction, fertilization success in in vitro fertilization (IVF) cycles for both conventional IVF (cIVF) and intracytoplasmic sperm injection (ICSI) is based on the assessment of pronuclei (PN) development and scoring.

Fertilization is the result of the fusion of a spermatozoon and an oocyte, which is confirmed by the presence of two nuclei in the oocyte: a female nucleus (or maternal pronucleus: m-PN) from the oocyte, and a male nucleus (or paternal pronucleus: p-PN) from the spermatozoon. Therefore, a normal fertilized zygote displays 2PN 16 to 18 h post insemination. Multiple PN zygotes (>2) are discarded due to an increased risk of aneuploidy. The most controversial question concerns 0 (no sign of fertilization) and 1PN (monopronuclear) zygotes. Indeed, embryos derived from this pronuclear stage are largely considered abnormal and unsuitable for transfer. When focusing on 1PN zygotes, several hypotheses have been suggested to explain the origin of this “defective” fertilization: parthenogenesis leading to haploidy and abnormal embryos, asynchronism of m-PN and p-PN formation, fusion of the two PN, or early pronuclear breakdown [1].

Although embryos derived from these types of zygotes have a normal morphology, the decision to use them or not varies considerably from one assisted reproductive technology (ART) center to another. Indeed, several studies showed that additional investigations could be required such as timelapse monitoring [2], preimplantation genetic testing (PGT) [1,2,3] or more morphometric parameters such as pronuclear areas and diameters [3]. Nevertheless, other groups routinely transfer 1PN embryos, achieving good development without further analysis [4].

In this retrospective study, we investigate embryo outcomes, live birth rates and neonatal outcomes in a cohort of 1PN zygotes in fresh and frozen–thawed cycles (cIVF and ICSI cycles) with comparison to 2PN-derived embryos. The first aim of our study is to share our experience with 1PN-derived embryos, in an attempt to answer the question posed by several teams and recently reviewed by M Kemper “what happens to abnormally fertilized embryos?” [5].

## 2. Materials and Methods

### 2.1. Study Design and Patients

**Figure 1:**
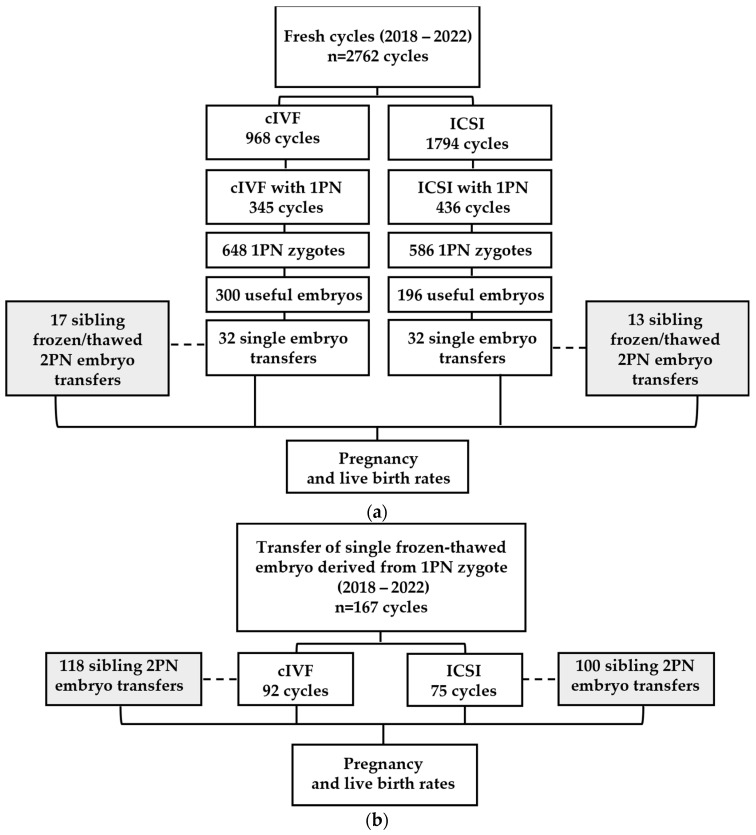
Analyzed cycles, 1PN zygotes’ evolution and embryo transfers in (**a**) fresh and (**b**) frozen–thawed cycles. The dashed lines show the control groups (gray square) corresponding to the transfer of frozen/thawed 2PN embryos in the same groups of patients who underwent a transfer of 1PN embryos.

This retrospective study was conducted at the Center for Reproductive Medicine of the University of Liège, located at the Citadelle Hospital in Belgium, between January 2018 and December 2022. A total of 2762 fresh cycles (n = 2022 patients) and 167 frozen–thawed cycles (n = 150 patients) were included. It should be noted that the embryos transferred in the frozen–thawed cycles were not necessarily derived from the 2762 fresh cycles previously mentioned. All patients underwent cIVF or ICSI cycles and selection was performed according to the presence of at least one 1PN zygote, as described in Figure 1a,b. Embryo and neonatal outcomes were studied for 648 and 586 1PN zygotes in cIVF and ICSI fresh cycles, respectively (Figure 1a). To interpret the results obtained with embryos derived from 1PN zygotes, a comparison was made within the same groups of patients with their surplus embryos derived from 2PN that were frozen and thawed as part of a frozen embryo transfer (FET) (gray square in Figure 1a,b). 

The study was approved by the Ethics Committee (number 2048) of the Citadelle Hospital of Liège, Belgium.

### 2.2. Ovarian Stimulation and Insemination

A total of 334 cycles in the cIVF group and 401 cycles in the ICSI group were carried out mainly using antagonist-controlled ovarian hyperstimulation protocols (in 96.8% and 92% of the cycles, respectively). Some of the other patients received a GnRH agonist protocol. Ovarian response to stimulation was monitored by hormonal blood tests and ultrasound assessments of follicular growth, as previously described [6]. Oocyte pick-ups were performed by transvaginal aspiration under ultrasound guidance around 36 h after human chorionic gonadotrophin (Ovitrelle^®^, 250 micrograms/0.5 mL, Merck, Rahway, NJ, USA) injection, and retrieved oocytes were then fertilized using cIVF or ICSI. 

The present study included treatments with partner or donor sperm. For patients in the cIVF group, donor sperm was used in 244 cycles (70.7%) and partner ejaculated sperm in 101 cycles (29.3%). In the ICSI group, donor sperm was used in 45 cycles (10.3%) and partner sperm in 391 cycles (89.7%) from either fresh or frozen samples. ICSI was applied in cases of poor sperm quality or failed cIVF in previous cycles.

### 2.3. Embryo Culture, Evaluation, Transfer and Cryopreservation

After fertilization, oocytes were allocated to culture for 2/3 or 5/6 days in 20 µL drops of Global^®^ medium (CooperSurgical, Trumbull, CT, USA) in dishes overlayed by Ovoil^TM^ (Vitrolife, Gothenburg, Sweden) [7]. For extended culture, medium was changed on day 3. Assessment of fertilization by evaluation of pronuclei (0, 1, 2 or >2PN) was performed 18 h post insemination (hpi) and followed by an early cleavage stage evaluation at 25 hpi. Oocytes showing more than 2PN were discarded and embryos derived from 0, 1 or 2PN zygotes were evaluated until day 2/3 or 5/6 of culture. According to our center’s protocol, a minimum of four fertilized oocytes was required for prolonged culture. In the case of a single oocyte, transfer was carried out on day 2 of culture if the embryo was of sufficient quality.

Cleavage- and blastocyst-stage embryos were assessed using our routine examination protocol based on Istanbul consensus criteria [8]. Cleaved embryos were classified considering blastomere number and symmetry, percentage of fragmentation and cytoplasmic appearance, as previously described [7]. Blastocyst quality was defined according to the degree of expansion, as well as the morphology of the inner cell mass and trophectoderm. Usable embryos were transferred and/or cryopreserved by vitrification. After embryo transfer, luteal phase was sustained by progesterone administration and pregnancy was defined by an hCG level greater than 100 UI/L fourteen days after embryo replacement. In case of pregnancy, hormonal supplementation was maintained until 8 weeks of gestation in fresh and frozen embryo transfer after a modified natural cycle (mNC) or until 12 weeks of gestation in a hormone replacement therapy (HRT) cycle.

### 2.4. Embryo Thawing

Frozen–thawed embryos were transferred either in HRT or mNC. Day 3 embryos were thawed one day before transfer, and day 5/6 embryos on the morning of transfer day. 

## 3. Results

The present study included two groups of patients selected between 2018 and 2022 (Figure 1). In the first group (Figure 1a) of 2762 fresh cycles (968 cIVF and 1794 ICSI cycles), 781 cycles with at least one 1PN zygote were identified (345 cIVF and 436 ICSI cycles). We focused our investigation on this cohort of 1234 1PN zygotes, of which 64 single fresh embryo transfers were performed. 

Within the same period, the second group (Figure 1b) comprised 167 transfers of frozen–thawed embryos derived from 1PN zygotes, previously obtained after either cIVF (n = 92) or ICSI cycles (n = 75). 

### 3.1. Study Population

For fresh cycles (Table 1), mean age of patients was 36.1 in the cIVF group (345 cycles performed in to 321 patients) and 34.3 years in the ICSI group (436 cycles performed in 410 patients), with a significant difference between the two groups (*p* < 0.001). Body mass index (BMI) as well as the mean cycle rank showed a significant difference between patient groups *p* < 0.01 and *p* < 0.001, respectively. Nevertheless, no differences were reported between the two groups regarding the number of retrieved oocytes or the hormonal status of E2 and P4. However, LH concentration on the day when the decision to induce ovulation was taken was significantly higher in the ICSI group in comparison to the cIVF group (*p* < 0.001), but without clinical relevance.

For frozen/thawed cycles (Table 2), the average patient age was 36.1 in the cIVF group (92 cycles performed on 79 patients) and 34.7 years in the ICSI group (75 cycles performed on 71 patients). Except differences in terms of fresh cycle ranks (*p* < 0.01) and numbers of retrieved oocytes (*p* < 0.05), all patient characteristics were comparable between the two groups. In the cIVF FET group, mNC was applied in 32.6% of the cycles, with the remaining 67.4% being HRT cycles. In the ICSI FET group, these figures were 40% and 60%, respectively.

### 3.2. Embryo Outcomes

In group 1, the fate of a total of 1234 1PN zygotes (648 cIVF and 586 ICSI) was analyzed. Forty-six percent of them gave rise to embryos of sufficient quality to be transferred or frozen (day 2, 3 or 5/6) in the cIVF group. However, this rate was significantly lower (33%) in the ICSI group (*p* < 0.001). In terms of embryo quality, no difference was observed between the two groups for either the A, B or C score. Nevertheless, blastulation rate was significantly higher in the cIVF group (44%) in comparison to the ICSI group (20%) (*p* < 0.001) (Table 3).

Embryo outcomes and transfer details for cleavage stage and day 5/6 embryos are presented in Supplementary Materials.

### 3.3. Pregnancy and Clinical Outcomes of Cleaved 1PN Embryos in Fresh and Frozen–Thawed Cycles

To interpret the results obtained with embryos derived from 1PN, a comparison was made within the same groups of patients using their own embryos derived from 2PN that were frozen and thawed as part of a frozen embryo transfer (FET). 

First regarding fresh 1PN embryo transfers (Group 1), PR and LBR in the cIVF group (n = 32) were similar to those obtained with surplus frozen–thawed 2PN embryos (n = 17) (Table 4). However, the difference was more notable in the ICSI group, with higher PR and LBR for frozen–thawed 2PN transfers (n = 13) as compared to fresh 1PN transfers (n = 32), reaching significance for PR (54% and 12.5%, *p* < 0.005). 

In contrast when analyzing results obtained for transfers with frozen–thawed 1PN embryos (n = 75), Group 2, as compared with their frozen–thawed 2PN counterpart (n = 100), no differences were found in the ICSI group, whereas the only tendency found in the IVF group was a non-significant increase in miscarriage rate (MR) for frozen–thawed 2PN transfers (n = 118) in comparison to 1PN (n = 92; MR 48% versus 29%, respectively, NS; Table 4).

A more detailed analysis of the 1PN embryos across the various groups revealed that the fresh single embryo transfers (32 in the cIVF group and 32 in the ICSI group) allowed seven pregnancies in the cIVF group (PR = 21.9%) as compared to four pregnancies in the ICSI group (PR = 12.5%). The seven cIVF pregnancies ended in five deliveries (LBR = 15.6%) of healthy newborns and two miscarriages (28.6%). In the ICSI group, one birth of a healthy newborn (LBR = 3.1%) and three miscarriages (75%) were observed. In frozen–thawed cycles, 36 pregnancies were achieved out of the 167 transfers. A non-significant difference was observed in pregnancy and live birth rates between embryos derived from cIVF cycles (PR = 26.1%, LBR = 18.5%) and ICSI cycles (PR = 16%, LBR = 10.7%). One pregnancy in the cIVF group delivered twins for a total of 18 babies in this group (Table 4).

### 3.4. Neonatal Outcome of Cleaved 1PN Embryos in Fresh and Frozen–Thawed Cycles

Neonatal data showed that eight births were achieved before 37 weeks of gestation and only one before 32 weeks of gestation of all births of our study. The gender proportion is balanced across the different 1PN embryos groups, with an overall sex ratio of 1.13 for the 32 births. However, birth weight was higher in the frozen–thawed cycles than in the fresh cycles without significance (Table 5).

The Non-Invasive Prenatal Test (NIPT), fully reimbursed in Belgium for all pregnancies, was performed on 18 patients at the late first trimester, showing no abnormalities for chromosomes 13, 18 or 21. Furthermore, no congenital anomalies were reported in any of the newborns.

## 4. Discussion

In our center, over a period of five years, embryo transfers using 1PN zygotes have resulted in the birth of 32 babies. The decision to transfer such embryos has been widely debated for several years in assisted reproduction groups, but no consensus has yet been reached. It was therefore decided to perform transfer of a fresh 1PN embryo when it was the only embryo available or, more anecdotally, when its quality was far superior to that of normally fertilized embryos (2PN).

Regarding embryo outcomes in fresh cycles, our analysis showed that the percentage of 1PN embryos reaching the blastocyst stage was almost twice as high in the cIVF group (44%) as in the ICSI group (20%). The same observation was reported by other studies: 21.4% versus 10.7% in the publication of Itoi [4], 26% versus 13.8% in the data reported by Bradley et al. [1] and 41.6% versus 23.25% in the study published by Li et al. [9]. As for the Araki study, the blastulation rate was 32.2% for 1PN-derived embryos but without distinction for the used insemination technique [3]. However, in a study analyzing the genome-wide haplotype of embryos derived from 0PN, 1PN and 2PN, the blastulation rate in 1PN ICSI embryos was higher than our results, at approximately 45.5% [10].

In this study, for the embryo outcomes analysis, we investigated the fate of 1PN embryos without comparison to 2PN embryos. Nevertheless, when such comparison is performed, it is confirmed that 2PN-derived embryos blastulate better than 1PN embryos [1,3,4,11]. This low potential for blastulation and production of good-quality embryos from 1PN zygotes are generally attributed to a chromosomal defect, particularly after ICSI rather than cIVF [12]. ICSI is the most effective assisted reproductive procedure for enabling fertilization in severe forms of male factor indications. Thus, the low blastulation rate in ICSI groups could be partly explained by sperm quality [13]. Mateo et al. had shown in 2013 by studying a small cohort of 54 embryos derived from 1PN ICSI zygotes that most of them were chromosomally abnormal [14]. The presence of 1PN zygotes after cIVF could be due to inappropriate timing of fertilization control. Indeed, it is documented that embryos development kinetics and pronuclei formation are different in zygotes arising from IVF in comparison to those from ICSI [15]. In addition, Lim et al. confirmed that the number of unipronuclear embryos was determined to be diploid following karyotyping and fluorescence in situ hybridization (FISH) [16].

It is also important to highlight that, in our results, a higher blastulation rate in the cIVF group compared to the ICSI group was observed in a population of significantly older patients (*p* < 0.001), confirming the efficacy of cIVF in cases of good sperm quality. 

To compare PR, MR, and LBR among the different groups of 1PN-derived embryos, we analyzed the fate of frozen 2PN embryos from the same patient cohorts which were then thawed and transferred. Despite the relatively small numbers in certain groups, we believe this allows for a more robust analysis with a comparable number of samples. To the best of our knowledge, such a comparison (as detailed in Table 4) has not been previously published by other teams. Therefore, direct comparisons with existing studies may be challenging. Nonetheless, we can investigate similarities between specific groups in our study and those reported in the literature. 

Generally, studies are controversial regarding the pregnancy rate and fate in fresh embryo transfer cycles compared to frozen embryo transfer cycles. While some studies show no differences between the two types of cycles, as reported by Gullo et al. [17], other teams have demonstrated a higher pregnancy rate in frozen cycles [18]. If we focus this part of the discussion only on the 1PN embryo groups, we observed that live birth rates (LBR) were slightly higher in frozen cycles (cIVF 18.5% and ICSI 10.7%) in comparison to fresh cycles (cIVF 15.6% and ICSI 3.1%). The same trend, but more marked, has already been reported by Li et al. with 32.1% for cIVF and 15.25% for ICSI in frozen cycles in comparison to 8% for cIVF and 0% for ICSI in fresh cycles [9]. Few studies have compared live birth rates after embryo transfer developed from 1PN in fresh and frozen–thawed cycles [5]. 

The comparison of 1PN embryos to their corresponding 2PN frozen–thawed counterparts showed no significant difference in terms of PR, MR, and LBR, except for the ICSI group, where the PR was significantly higher for frozen–thawed 2PN embryos compared to fresh 1PN embryos (54% vs. 12.5%, respectively). This difference was maintained for LBR but is not statistically significant (15% vs. 3.1%, respectively). If we consider only the frozen–thawed cycles from our study, we observed that the PR and LBR rates were not statistically different between 1PN and 2PN embryos in both cIVF and ICSI cycles. Itoi et al. conducted a similar comparison in 2015, but they transferred only three blastocysts in the ICSI 1PN group, which did not result in any pregnancies [4]. In an another study by Li et al. 2020, comparing vitrified-warmed embryos 1PN to 2PN but only in cIVF, no difference was noted for PR (41% vs. 46%, respectively) and LBR (27% vs. 35%, respectively) [9]. In this last study, the number of patients was considerably higher compared to ours, especially for the 2PN control group. One of the limitations of our study is the relatively small number of patients in the eight groups analyzed. Nevertheless, we compare 1PN embryos to 2PN embryos from the same groups of patients, which is a particular strength of our study. 

There were, in total, 32 live babies born after 1PN-derived embryo transfer without difference in gender balance between the different groups, with a global sex ratio (male/female) of 1.13. Few publications investigating neonatal outcomes after 1PN embryo transfer with sex and gender data are available. However, the study by Li et al. showed that the sex ratio was also not significantly different between the 1PN and 2PN groups in cIVF cycles [9]. The investigation of neonatal outcomes showed a slightly elevated birth weight in the frozen–thawed 1PN group in comparison the fresh cycles group. Such difference between fresh and frozen cycles was previously documented in several studies and reviews [17,19,20,21]. 

Decision to transfer 1PN embryos or not has evolved over the last few decades. Indeed, less recent studies analyzing the chromosomal composition of embryos derived from 1PN zygotes suggest that these embryos should not be used for transfer or cryopreservation after IVF or ICSI treatment [14,22,23,24]. Nevertheless, more recently, the clinical use of these embryos has been reconsidered thanks to the development of prolonged culture at the blastocyst stage, the time-lapse technique and PGT-A [1,2,3,10,25,26,27]. Regarding congenital malformation, it is well documented, as recently reviewed by Veeramani et al. in 2024, that newborns conceived using assisted reproduction techniques seem to present a higher risk of congenital anomalies, particularly with the ICSI technique, compared with newborns conceived naturally [28]. However, in our study, single transfer of 1PN embryos has resulted in the birth of 32 healthy babies. 

In the present study, decision to transfer each 1PN embryo, instead of available 2PN embryos, was based on its capacity to develop to the blastocyst stage with sufficient morphological quality. This kind of selection was previously chosen by Itoi and Li’s teams [4,9,29]. Transfer of day 2/3 embryos derived from 1PN zygotes was only performed in the absence of normally fertilized embryos.

## 5. Conclusions

In conclusion, we observed better outcomes for 1PN zygotes in cIVF cycles compared to ICSI cycles. Our center’s policy of transferring good-quality 1PN-derived embryos has resulted in the birth of 32 healthy babies, which may increase the chances of pregnancy in some patients. Given these results, the elimination of 1PN zygotes should perhaps not be automatic but based on their blastocyst development and quality. 

## Figures and Tables

**Table 1:** Study population of patients with at least 1PN zygote in fresh cycles. Hormonal dosages were performed on the day of the decision to induce ovulation, i.e., on the day of induction or 1–2 days before.

	cIVF n = 321 Patients	ICSI n = 410 Patients	*p* Value
Age	36.15 ± 4.6	34.36 ± 5.3	*p* < 0.001
BMI	24.3 ± 4.1	25.3 ± 5	*p* < 0.01
Cycle rank	1.2 ± 0.5	1.6 ± 1.1	*p* < 0.001
No of retrieved oocytes	12 ± 7.6	11.4 ± 7.8	NS
Serum [E2] pg/mL	2104 ± 1431	2255 ± 1875	NS
Serum [P4] ng/mL	0.75 ± 0.51	0.75 ± 0.5	NS
Serum [LH] mUI/mL	1.9 ± 1.9	2.6 ± 3.4	*p* < 0.001

NS: non-significant.

**Table 2:** Study population of patients with at least 1PN zygote in frozen–thawed cycles.

	cIVF n = 79 Patients	ICSI n = 71 Patients	*p* Value
Age	36.1 ± 5	34.7 ± 5.8	NS
BMI	24.6 ± 11.8	25.5 ± 11.9	NS
Fresh cycle rank *	1.2 ± 0.6	2.2 ± 1.5	*p* < 0.01
No of retrieved oocytes	15.3 ± 9	12.3 ± 8.6	*p* < 0.05
Embryo transfer rank **	2.2 ± 1.5	2.2 ± 1.4	NS

* Correspond to the fresh cycle rank of frozen embryos. ** Correspond to the rank of frozen/thawed embryo transfer. NS: non-significant.

**Table 3:** Embryo outcomes per group in fresh cycles (Group 1). Usable embryos correspond to transferred and/or frozen embryos.

	cFIV n = 648 1PN Zygotes	ICSI n = 586 1PN Zygotes	*p* Value
Usable embryos (cleavage stage and D5–6)	300/648 (46.3%)	196/586 (33.4%)	*p* < 0.001
Score A	103/300 (34.3%)	67/196 (34.2%)	NS
Score B	90/300 (30%)	45/196 (22.9%)	NS
Score C	107/300 (35.7%)	84/196 (42.9%)	NS
Transferred embryos	52/648 (8%)	55/586 (9.4%)	NS
Discarded embryos	348/648 (53.7%)	390/586 (66.6%)	*p* < 0.001
Blastulation rate	195/439 (44.4%)	66/329 (20.1%)	*p* < 0.001

NS: non-significant.

**Table 4:** Pregnancy and clinical outcomes of 1PN embryos in fresh and frozen–thawed cycles were compared to 2PN FET in the same groups of patients. Pregnancy rate (PR), miscarriage rate (MR) and live birth rate (LBR). * *p* < 0.05.

	Fresh cIVF (n = 32)		Fresh ICSI (n = 32)		Frozen–Thawed Cycles cIVF (n = 92)		Frozen–Thawed Cycles ICSI (n = 75)	
	1PN Fresh (n = 32)	2PN FET (n = 17)	1PN Fresh (n = 32)	2PN FET (n = 13)	1PN FET (n = 92)	2PN FET (n = 118)	1PN FET (n = 75)	2PN FET (n = 100)
Pregnancy rate	7/32 (21.9%)	4/17 (24%)	4/32 (12.5%)	7/13 (54%) *	24/92 (26.1%)	27/118 (23%)	12/75 (16%)	27/100 (27%)
Miscarriage rate	2/7 (28.6%)	1/4 (25%)	3/4 (75%)	5/7 (71%)	7/24 (29.2%)	13/27 (48%)	4/12 (33.3%)	11/27 (41%)
Live birth rate	5/32 (15.6%)	3/17 (18%)	1/32 (3.1%)	2/13 (15%)	17/92 (18.5%)	14/118 (12%)	8/75 (10.7%)	15/100 (15%)

In addition to the absolute values represented in the table, the percentage was calculated for each group.

**Table 5:** Neonatal outcomes in cIVF and ICSI groups of cleaved 1PN embryo in fresh and frozen–thawed cycles compared to 2PN frozen–thawed embryo in the same groups of patients.

	Fresh cIVF		Fresh ICSI		Frozen–Thawed cIVF Cycles		Frozen–Thawed ICSI Cycles	
	1PN Fresh (n = 5)	2PN FET (n = 3)	1PN Fresh (n = 1)	2PN FET (n = 3) ^●^	1PN FET (n = 18)	2PN FET (n = 14)	1PN FET (n = 8)	2PN FET (n = 16) ^●●^
Weeks of gestation	38 ± 1.5	38.5 ± 0.5	39.5	36.2 ± 2.9	37.8 ± 2.7	38.5 ± 1.4	38.3 ± 1.5	38.7 ± 1.6
Gender	2♀/3♂	1♀/2♂	1♂	1♀/2♂	10♀/8♂	8♀/6♂	3♀/5♂	10♀/6♂
Birth weight (g)	2743 ± 478	4205 ± 398	2950	2611 ± 1072	3336 ± 564	3336 ± 524	3581 ± 341	3259 ± 508
Average length (cm)	46.9 ± 2.8	52.7 ± 1.5	49	47.5 ± 3.8	50 ± 3	50 ± 2.2	48 ± 6	45.6 ± 13

^●^ 3 babies for 2 live births. ^●●^ 16 babies for 15 live births. ♀: female; ♂: male.

## Data Availability

Data available on request due to restrictions of privacy. The data presented in this study are available on request from the corresponding author.

## Supplementary Materials

The following supporting information can be downloaded at: https://www.mdpi.com/article/10.3390/medicina60081361/s1, Supplementary Table S1: Useful embryo proportion according to embryo stage and quality. Supplementary Table S2: Transferred embryo proportion according to embryo stage and quality.
