# Supplementary material for: Birth of Thirty-Two Healthy Babies Following Transfer of Fresh and Frozen–Thawed Embryos Derived from Monopronuclear Zygotes: A Retrospective Study
Source: Medicina (Kaunas). 2024 Aug 21;60(8):1361. doi: 10.3390/medicina60081361 (PMC11355993; doi:10.3390/medicina60081361)
Supplement: Supplementary file 1 [file medicina-60-01361-s001.zip › medicina-3144025-supplementary.pdf]

Supplementary Table S1: Useful embryo proportion according to embryo stage and quality.

	Useful embryos		
	Score A	Score B	Score C
**cIVF n=300**			
Cleavage stage embryos n=105 (35%)	30 (28.6%)	25 (23.8%)	50 (47.6%)
Blastocysts n=195 (65%)	73 (37.4%)	65 (33.3%)	57 (29.3%)
**ICSI n=196**			
Cleavage stage embryos n=130 (66%)	48 (36.9%)	28 (21.5%)	55 (41.6%)
Blastocysts n=66 (34%)	19 (28.8%)	17 (25.7%)	30 (45.5%)

Supplementary Table S2: Transferred embryo proportion according to embryo stage and quality.

Transferred embryos			
**cIVF n=52**		**ICSI n=55**	
Cleavage stage	*Blastocysts*	*Cleavage stage*	*Blastocysts*
42 (80%.7)	10 (19.3%)	52 (94.5%)	3 (5.5%)
